# Dapsone-Induced Methemoglobinemia: A Case Report

**DOI:** 10.7759/cureus.22466

**Published:** 2022-02-21

**Authors:** Monika Shenouda, Miriam Padilla, Juan Silva, Hector Castillo, Armaity Austin

**Affiliations:** 1 Family Medicine, California Hospital Medical Center, Los Angeles, USA

**Keywords:** saturation gap, methylene blue, cyanosis, dapsone, methemoglobinemia

## Abstract

Here, we present the case of a 64-year-old male with a rare cause of cyanosis due to dapsone-induced methemoglobinemia who was treated successfully with methylene blue and high-dose Vitamin C. Our case emphasizes the importance of history taking, knowledge, and high index of suspicion of drug-induced methemoglobinemia, especially in the presence of saturation gap. This condition can be fatal if left untreated.

## Introduction

Methemoglobinemia is a rare and potentially life-threatening condition characterized by the decreased oxygen-carrying capacity of hemoglobin due to the conversion of iron from the reduced ferrous (Fe^2+^) state to the oxidized ferric (Fe^3+^) state, which makes it incapable of binding oxygen molecules [1,2]. Cyanosis occurs when 10-25% of the total hemoglobin turns into methemoglobin [3]. Methemoglobinemia can be congenital; however, most common causes, though rare, are acquired due to chemical or topical agents. In our case, it was dapsone. If a patient’s history confirms exposure to an oxidative agent, the patient should be treated immediately to prevent adverse outcomes [4]. Due to the rarity of this condition, it is important for clinicians to recognize the symptoms and offending agents of methemoglobinemia for prompt diagnosis and treatment. Here, we report the diagnosis and management of a patient with dapsone-induced methemoglobinemia with evident cyanosis.

## Case presentation

**Figure 1:**
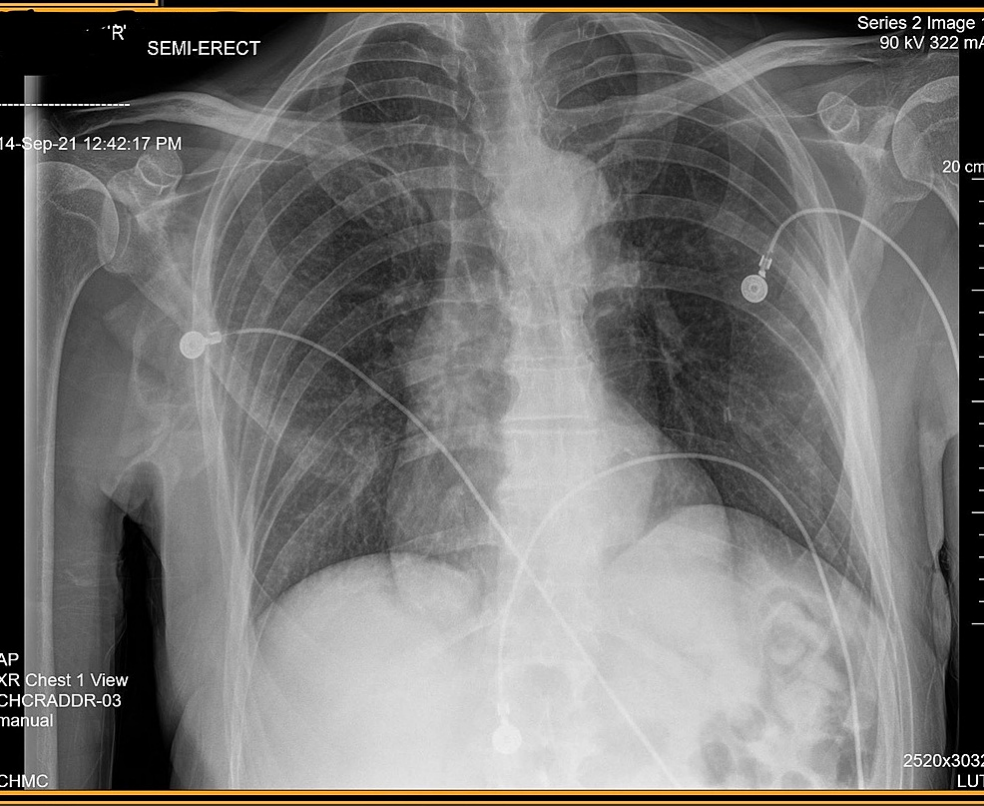
Single front-view X-ray of the chest with no evidence of acute disease and a calcified aorta consistent with atherosclerotic disease.

A 64-year-old Hispanic male with a history of oral and esophageal ulcers was started on dapsone 100 mg daily after an extensive workup for suspected Behcet’s syndrome. Three weeks later, he presented to the emergency department with progressively worsening shortness of breath, ataxia, generalized body aches, headaches, and palpitations. On presentation, he was found to have tachypnea in the 40s with retractions and marked peripheral and central cyanosis. Oxygen saturation on room air was 74%. He was placed on supplemental oxygen via a high-flow nasal cannula. Arterial blood gases (ABGs) were significant for pH of 7.70, pCO_2_ of 17, PaO_2_ of 328, SaO_2_ of 97.8%, and methemoglobin of 4 (Table 1). A chest X-ray showed no acute cardiopulmonary disease without pleural effusion or pneumothorax (Figure 1). Given the discrepancy of low SpO_2_ and normal SaO_2_ and PaO_2_ on ABG analysis, a diagnosis of dapsone-induced acquired methemoglobinemia was considered. He was admitted to the intensive care unit on the maximum setting of a high-flow nasal cannula. The offending agent was discontinued. He was treated with methylene blue 1 mg/kg intravenously daily and high-dose vitamin C 5 g intravenously every six hours. ABGs were monitored every 12 hours to trend PaO_2_ and methemoglobin levels. He continued to show signs of improvement with progressively decreasing oxygen requirements. He was discharged one week later on a 2 L nasal cannula, was ambulating without any difficulty, and was maintained on close outpatient follow-up with an uneventful recovery.

**Table 1:** Arterial blood gas monitoring during hospitalization.

	Hospital Day #4	Hospital Day #3	Hospital Day #2	Hospital Day #1
pH	7.45	7.48	7.61	7.70
pCO_2_	35	29	20	17
pO_2_	83	135	170	328
HCO_3_	24.3	21.6	20.1	20.1
O_2_ saturation	97.5	96.9	96.7	97.8
Methemoglobin	1.9	2.5	2.5	4.0
PaO_2_/FiO_2_	395	338	425	328

## Discussion

Methemoglobin forms due to the conversion of iron from the reduced ferrous (Fe^2+^) state to the oxidized ferric (Fe^3+^) state. The oxidized state of iron does not bind to oxygen, and the presence of the ferric state (Fe^3+^) shifts the oxygen dissociation curve to the left. This shift allows ferrous iron (Fe^2+^) to increase affinity for oxygen, resulting in decreased oxygen release to the tissues [5]. Normally, a small amount of iron oxidizes into the ferric state during the normal delivery of oxygen to the tissues. Our bodies can maintain decreased methemoglobin levels through the actions of cytochrome b5 reductase, which utilizes nicotinamide adenine dinucleotide hydrogen to reduce methemoglobin back to normal hemoglobin. Another pathway is by utilizing nicotinamide adenine dinucleotide phosphate (NADPH)-methemoglobin reductase. NADPH is formed through glucose-6-phosphate dehydrogenase (G6PD) in the hexose monophosphate shunt. The NADPH pathway normally contributes very little to reduction, but under oxidative stress, it can be enhanced.

Dapsone has anti-inflammatory, antibacterial, and immunosuppressive properties and is used in a wide variety of medical conditions, such as leprosy, dermatitis herpetiformis, autoimmune bullous dermatoses, malaria, and *Pneumocystis jirovecii* infections. Dapsone has hydroxylamine derivatives that induce severe oxidative stress to the hemoglobin inside the erythrocytes. Dapsone is among the offending drugs that cause acquired methemoglobinemia alongside nitrite and nitrate derivatives, sulfonamides, phenazopyridine, and some anesthetics and antimalarials [6]. Clinical symptoms with cyanosis out of proportion to respiratory distress are key to diagnosing methemoglobinemia. The characteristic finding of cyanosis with low SpO_2_ but with normal levels of PaO_2_ on ABG analysis. Because methemoglobin does not affect oxygen delivery to the blood plasma in the alveoli, PaO_2_ remains unaffected.

Initial management of patients with methemoglobinemia is the discontinuation of the offending agent. For patients, with methemoglobin levels exceeding 30% or signs of hypoxia, administration of methylene blue intravenously at 1 to 2 mg/kg is often required [7]. Treatment with methylene blue is contraindicated in cases with concurrent G6PD deficiency and in individuals taking serotonergic agents due to the risk of serotonin syndrome. Methylene blue has been shown to be a potent monoamine oxidase inhibitor [8]. Alternative treatments include hyperbaric oxygen, exchange transfusions, activated charcoal, or high-dose vitamin C [8,9].

## Conclusions

Methemoglobinemia is a rare cause of hypoxia and cyanosis. It can be a potentially fatal disease if not addressed in a timely manner or left untreated. Our case emphasizes the importance of history taking, knowledge, and a high index of suspicion of drug-induced methemoglobinemia, especially in the presence of a saturation gap. Methemoglobin levels are available on modern ABG analysis and aid in appropriate and timely diagnosis. Methylene blue is the recommended treatment, with alternative treatments including high-dose vitamin C, hyperbaric oxygen, exchange transfusions, or activated charcoal. Clinicians should be aware of the signs and symptoms of methemoglobinemia and include them in the differential diagnosis.
